# Fuel-cell parameter estimation based on improved gorilla troops technique

**DOI:** 10.1038/s41598-023-35581-y

**Published:** 2023-05-29

**Authors:** Abdullah Shaheen, Ragab El-Sehiemy, Attia El-Fergany, Ahmed Ginidi

**Affiliations:** 1 Department of Electrical Engineering, Faculty of Engineering, Suez University, Suez, 43533 Egypt; 2 Department of Electrical Engineering, Faculty of Engineering, Kafrelsheikh University, Kafrelsheikh, 33516 Egypt; 3 Electrical Power and Machines Department, Faculty of Engineering, Zagazig University, Zagazig, 44519 Egypt

**Keywords:** Fuel cells, Energy science and technology, Engineering

## Abstract

The parameter extraction of the proton exchange membrane fuel cells (PEMFCs) is an active study area over the past few years to achieve accurate current/voltage (I/V) curves. This work proposes an advanced version of an improved gorilla troops technique (IGTT) to precisely estimate the PEMFC’s model parameters. The GTT's dual implementation of the migration approach enables boosting the exploitation phase and preventing becoming trapped in the local minima. Besides, a Tangent Flight Strategy (TFS) is incorporated with the exploitation stage for efficiently searching the search space. Using two common PEMFCs stacks of BCS 500W, and Modular SR-12, the developed IGTT is effectively applied. Furthermore, the two models are evaluated under varied partial temperature and pressure. In addition to this, different new recently inspired optimizers are employed for comparative validations namely supply demand optimization (SDO), flying foxes optimizer (FFO) and red fox optimizer (RFO). Also, a comparative assessment of the developed IGTT and the original GTT are tested to ten unconstrained benchmark functions following to the Congress on Evolutionary Computation (CEC) 2017. The proposed IGTT outperforms the standard GTT, grey wolf algorithm (GWA) and Particle swarm optimizer (PSO) in 92.5%, 87.5% and 92.5% of the statistical indices. Moreover, the viability of the IGTT is proved in comparison to various previously published frameworks-based parameter's identification of PEMFCs stacks. The obtained sum of squared errors (SSE) and the standard deviations (STD) are among the difficult approaches in this context and are quite competitive. For the PEMFCs stacks being studied, the developed IGTT achieves exceedingly small SSE values of 0.0117 and 0.000142 for BCS 500 and SR-12, respectively. Added to that, the IGTT gives superior performance compared to GTT, SDO, FFO and RFO obtaining the smallest SSE objective with the least STD ever.

## Introduction

Due to a number of important factors, including the decline of conventional fuels, the trend of environmental concerns, and the rise in their price, clean energy sources have attracted significant interest around the world^1^. The fuel cell (FC), which can transform the chemical energy form into electrical form via chemical processes, is a reliable source of clean energy^2^. Even though there are myriads of FC types in the industry, Proton exchange membrane (PEM) FCs (PEMFCs) offer notable qualities such as low operating pressure and temperature, no wasted materials, and a high-efficiency level. Depending on the operating conditions, the regular temperature level ranges from 50 to 100 °C, and its efficiency is between 30 and 60%^3^. Over the past few decades, modeling of PEMFCs has attracted a lot of interest^4^. The major goal is to create a PEMFC’s model that is accurate, useful for software simulations, and closely resembles experimental models. Time and effort can be saved in this manner.

Each of these models has its own unique mathematical formulations, which include some unidentified parameters that are not displayed in the datasheets of manufacturer that are required for building an effective and reliable model^5^. Accordingly, myriads of handling methodologies was manifested to properly recognize the unidentified PEMFCs parameters such as adaptive filter-based^6^, electrochemical impedance spectroscopy-based techniques^7,8^, current switching methods^9^, and black box-based approaches^10^. However, these conventional optimizers are not commonly utilized to attain the adequate parameters of PEMFCs as they are inflexible and hard to implement. An electrochemical model that Mann et al.^11^ has developed in a semi-empirical formulation considering the steady state operation to emulate the PEMFC’s electrical characteristics. Mann's model has gained widespread acceptance over the last two decades for its ability to predict the PEMFC’s polarization properties with varied operating situations. However, Mann's unidentified parameters are tightly coupled and dramatically vary according to the load conditions which makes the model a nonlinearity issue. As a result, building the model using the aforementioned methods has become time-consuming and more complex^12^.

Recently, metaheuristic optimization methods have been used by many researchers to derive the PEMFC’s model required parameters owing to the major improvement of artificial intelligent-based methodologies. Metaheuristic techniques are the most effective and reliable method to use when estimating PEMFCs parameters as treated as one of optimization problems^4,12,13^. Regarding the optimization techniques used in this context, shark smell technique^14^, coyote optimization algorithm^15,16^, grey wolf algorithm (GWA)^17^, whale optimization algorithm (WOA)^18^, grasshopper optimization algorithm (GOA)^19^ bald eagle search optimizer^20^ and bonobo algorithm^21^. Besides, manta ray forage optimization (MRFO)^22^, pathfinder algorithm^23^, chaotic Harris hawk algorithm^24^, jellyfish searching algorithm^25^ and black widow algorithm^26^ were used to address the same problem. In addition to that, artificially ecosystem optimization^27^, tree-seed algorithm (TSA) and neural network algorithm^28^ have been applied for the same issue. Also, the researchers utilized the similar context of tree-growth algorithm^29^, flower pollination method^30^, political optimization algorithm, marine predator technique^31^ and slime mould optimization algorithm^32^ for parameter identification of PEMFCs. In^33^, a combination between teaching learning based optimizer and DE approach has been developed while a modified salp swarm optimizer has been presented to identify the optimal PEMFCs stack parameters in^34^.

Despite the benefits of self-adaptive nature optimizers, the optimizers still in necessity for improving time load, statistical analysis, and convergence rate. Accordingly, this article characterizes a Gorilla Troops technique (GTT)^35,36^ that uses a gorilla approach to extract the FC parameters properly. The GTT depends on several distinct behaviors of the gorillas that are mathematically simulated. In this context, five regarding behaviors are considered including traveling to other gorillas, migration to a strange region, competing for adult females, escorting the silverback, and migration toward a specified spot. These five behaviors are divided into two stages. In the exploratory stage, dual implementation of the migration approach is adopted enables for preventing becoming trapped in the local minima. At first, migration to an uncharted location to boost GTT searching capacity. Secondly, another migration approach is adopted towards a known place that greatly improves the GTT's capacity to look for various optimization spaces. Thirdly, a strategy is modeled to improve the equilibrium between investigation and exploitation by moving to the other gorillas. Additionally, the exploitation stage employs the use of two strategies, which greatly improves search efficacy. This work develops a GTT to precisely estimate the PEMFC’s model parameters. The double execution of the mutation approach in the created GTT enables for boosting the exploitation phase and preventing becoming trapped in the local minima. The conventional GTT has been successfully implemented in solving different engineering optimization issues. In^37^, GTT has been utilized for optimal tunning of a cascaded controller with type of Proportional Integral (PI)-Fractional Order PID to stabilize the frequency response of a microgrid with two-area power systems. In^38^, GTT has been developed in power networks for handling the optimal power flow. In^39^, GTT has been carried out on electrical distribution networks for the allocations of different types of distributed energy sources considering their probabilistic nature simultaneously with the loading uncertainties. In^40^, GTT has been employed on electric power networks to enhance the whole network performance with the addition of Thyristor-Controlled Series Capacitor compensators. Based on these successful implementations of the GTT and its magnificent merits of simplicity, ease of implementation, and speed of convergence, this work develops a distinctive GTT to accurately predict the PEMFC’s model parameters. The GTT has been successfully applied using two practical PEMFCs modules of BCS 500W and Modular SR-12. They are also examined at various pressures and temperatures. Additionally, several new recently motivated optimization techniques are used for comparison validation, including supply demand optimizer (SDO), Flying Foxes Optimizer (FFO) and Red Fox Optimizer (RFO).

The main points of the article are summarized as follows: (i) an advanced Improved GTT (IGTT) with a Tangent Flight Strategy (TFS) is efficiently developed for optimal PEMFCs parameters estimation considering two common industrial modules of BCS 500W, and Modular SR-12, (ii) The proposed IGTT is precisely applied for optimal diagnostics of FC modules with varying P_H2_/P_O2_ and temperature levels, (iii) The proposed IGTT provides higher accuracy and robustness compared with recently employed techniques of SDO, FFO, and RFO, and (iv) The outcomes and statistical assessments manifest the proposed IGTT superiority compared with several previously reported results which demonstrate its promising features in defining the PEMFC’s model parameters.

The rest of this article can be arranged as follows: the model of PEMFCs is elaborated in "Model of PEMFCs", whilst "Proposed methodology" illustrates the GTT plus problem formulation. In "Simulation results and discussion", the simulation, and discussion of the GTT’s results and comparisons are revealed when applied to PEMFCs stack for parameters extraction, whereas the main conclusion of the article and future extension are given in "Conclusion".

## Model of PEMFCs

### PEMFCs stack operation

**Figure 1:**
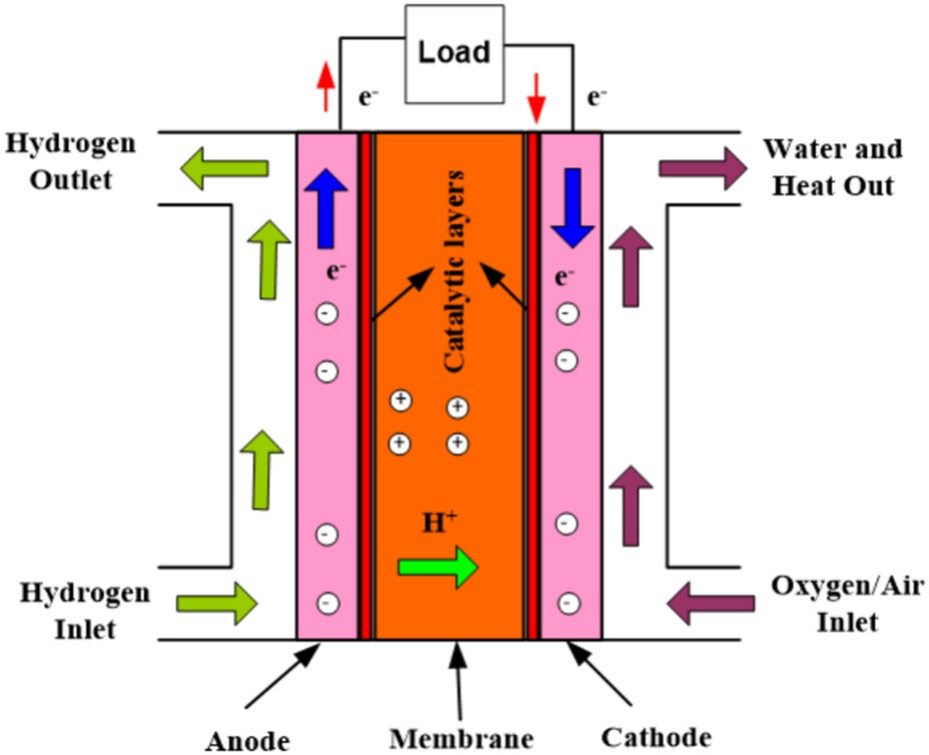
Fuel cell model.

In FCs, hydrogen and oxygen gases could be employed as a sustainable fuel for generating electrical current depending on chemical processes. In these stacks, a positive anode and a negative cathode are separated by the electrolyte. The FC anode and cathode, respectively, would receive the hydrogen and oxygen directly. As indicated in Fig. 1^22^, protons shall flow in a transversal manner along the short internally route after electrons are created because of a sufficient catalyst moving towards the cathode to supply the necessary electrical energy to the load in the outward channel. Heat is released during the chemical process that creates pure water.

Anode side:

1 $${H}_{2}\to 2{H}^{+}+2{e}^{-}$$

Cathode side:

2 $${O}_{2}+4{e}^{-}\to 2{O}^{-}$$

Total chemical reaction:

3 $${H}_{2}+\frac{1}{2}{O}_{2}\to {H}_{2}O+Electric current+Temperature rising$$

### Mathematical model of PEMFCs

To form the PEMFC’s model, the I-V characteristics (Polarization curves) could be mathematically displayed. The steady state performance of the PEMFCs is described using the electrochemical simplified model presented by Mann et al.^11^. This paradigm is widely employed in numerous literature analyses. The mathematical model for the stack’s output voltage (${V}_{stack}$) as illustrated in (4), which comprises of several series-connected cells (${N}_{cells}$)^11,19,41,42^.

4 $${V}_{stack}={N}_{cells}.({E}_{Nernst}-{v}_{act}-{v}_{\Omega }-{v}_{conc})$$

whereas *v*_*act*_ refers to the cell activation overpotential, ${E}_{Nernst}$ is the Nernst voltage per cell, ${v}_{conc}$ indicates the concentration over-potential, and ${v}_{\Omega }$ refers to the cell ohmic drop in voltage. The voltage ${E}_{Nernst}$ can be calculated using Eq. (2) under a reference temperature of 25 °C. Thus, these three voltages drop are provided as depicted in Eqs. (5)–(8)^11,41^.

5 $${E}_{Nernst}=-0.85\left({T}_{fc}-298.15\right)/1{0}^{3}+4.3085/1{0}^{5}\times {T}_{fc}\mathit{ln}\left({P}_{{H}_{2}}\sqrt{{P}_{{O}_{2}}}\right)+1.229$$

$${v}_{act}=-\left[{\xi }_{1}+{T}_{fc}\left({\xi }_{2}+{\xi }_{3}\mathit{ln}({C}_{{O}_{2}})+{\xi }_{4}\mathit{ln}({I}_{fc})\right)\right]$$

6 $${\mathrm{where }C}_{{O}_{2}}=\frac{{P}_{{O}_{2}}}{5.08\times 1{0}^{6}}.\mathit{exp}\left(\frac{498}{{T}_{fc}}\right)$$

$${v}_{\Omega }={I}_{fc}({R}_{m}+{R}_{c});{R}_{m}={\rho }_{m}l/{M}_{A}$$

7 $${\mathrm{where }\rho }_{m}=\frac{181.6\left[1+0.03{I}_{fc}/{M}_{A}+0.062{\left({T}_{fc}/303\right)}^{2}{\left({I}_{fc}/{M}_{A}\right)}^{2.5}\right]}{\left[\lambda -0.634-3{I}_{fc}/{M}_{A}\right].\mathit{exp}\left[4.18\times \left(({T}_{fc}-303)/{T}_{fc}\right)\right]}$$

8 $${v}_{conc}=-\beta .\mathit{ln}\left(1-J/{J}_{max}\right)$$

where ${P}_{O2}$ and ${P}_{H2}$ illustrate the regulating pressures of oxygen (*O*_*2*_) (atm) and hydrogen (*H*_*2*_), respectively, while $T_{fc}$ represents the working temperature of the FC (*K*). Moreover, ${C}_{O2}$ manifests the concentration of *O*_*2*_ (mol/cm^3^), *M*_*A*_ signifies the membrane area (*cm*), whereas ${I}_{fc}$ is its current (*A*) and *ξ*_*1*_ − *ξ*_4_ characterizes semiempirical coefficients^3,41^. Besides, *l* indicates the thickness of membrane (cm), whilst *R*_*c*_ and *R*_*m*_ reveal the leads and the membrane ohmic resistances (Ω); respectively. In addition to this, ${\rho }_{m}$ demonstrates the membrane resistivity (Ω.cm), *β* is handled as an empirical constant, and *λ* is treated as a changeable parameter, whilst *J*_*max*_ and *J* describe the maximum and actual thermal current densities (A/cm^2^), respectively^3,19^.

9 $$\beta =\wp .{T}_{fc}/2\alpha F$$

where *F,* $\wp$ and *α* represent Faraday’s, ideal gas constants, and charge transfer coefficient, respectively. It becomes clear that the temperature and current density can affect the concentrating voltage drop in linear relation based on a thorough understanding of Eqs. (5) and (6). To illustrate, at higher cell temperatures and larger current densities, the concentration polarization voltage is projected to be increased^11,41^. It can be deduced that the seven parameters are fundamentally approximated to construct an appropriate PEMFC’s model.

## Proposed methodology

### Problem formulation and description

Due to a lack of manufacturer data, the PEMFC’s modelling has significant non-linear characteristics and various undetermined parameters. This signifies that creating an accurate model will be incredibly challenging. Seven parameters should be calculated. The optimization objective (*FCF*), in this purpose, is stated as the minimization of the sum squared error between the experimental FC voltage and estimated model voltage. Thus, the PEMFCs parameters estimation problem is approached as an objective target. The issue in the present work may be thought of as a non-convex optimization issue. The *FCF* is written in Eq. (10) as follows^19,43^.

10 $$FCF=Min(SSE)=Min\left({\sum }_{m=1}^{{N}_{samples}}{\left[{V}_{FC,exp}\left(m\right)-{V}_{FC,est}\left(m\right)\right]}^{2}\right)$$

where *m* expresses the iteration counter, ${N}_{samples}$ designates the number of measured voltage data, ${V}_{FC,est}$ manifests the FC estimated calculated voltage, and ${V}_{FC,exp}$ represents the measured output voltage of the model. The optimization objective is restricted with inequality constraints for unknown seven parameters which are the minimum and maximum limits of these parameters. The GTT is applied to optimize these seven unknown parameters which are namely, *λ*, *ξ*_*1*_ − *ξ*_*4*_, *R*_*c*_, and *β* that obtain the best value of the SSE.

### Gorilla troops technique

The GTT depends on several distinct behaviors of the gorillas that are mathematically simulated. Five behaviors are taken into account in this situation to optimize gorilla behavior: three for the exploration stage and two for the exploitation stage. These activities include migration to a strange region, traveling to other gorillas, migration toward a specified spot, competing for adult females and escorting the silverback. Two stages represent these strategic options that can be divided into the exploitation stage and exploration stage as will be manifested in the following subsections.

#### Exploration stage

Three distinct behaviors, in this stage, are elaborated: the first one is to manifest GTT exploration (which is movement to an unidentified end point), whereas the second tactic represents the traveling behavior to other gorillas. Furthermore, the third tactic aims at encouraging GTT competences in determining a myriad of calculation spaces that represents the migration toward a specified spot. Equation (11) can represent these three behaviors mathematically, where the movement to unidentified end point tactic, in this equation, is selected if a random number (*rn)* is smaller than a factor (*Fr*). Besides, the traveling to other gorillas or migration toward a specified is carefully selected if a random number equals/ (is more than) 50%.

11 $$GtX(Itn+1)=\left\{\begin{array}{l}LB+r{n}_{1}\times (UB-LB),Fr>rn,\\ Z\times X(Itn)\times Q+{X}_{r}(Itn)\times (r{n}_{2}-D\times \left(1-Itn/MxItn\right)),0.5\le rn,\\ X(Itn)+(X(Itn)-Go{X}_{r}(t)))\times r{n}_{3}-((X(Itn)-Go{X}_{r}(Itn)\times {Q}^{2}),0.5>rn\end{array}\right.$$

12 $$D=\mathit{cos}(2\times r{n}_{4})+1,$$

13 $$Q=D\times \left(1-Itn/MxItn\right)$$

14 $$Z=[-(D\times \left(1-Itn/MxItn\right)),D\times \left(1-Itn/MxItn\right)].$$

where *rn*, *rn*_*1*_, *rn*_*2*_, *rn*_*3*_, and *rn*_*4*_ illustrate random values among [0, 1], whilst *X(Itn)* and *GtX(Itn* + *1)* define the full and forthcoming vectors of the gorilla's position. The arbitrary assignable variables *X*_*r*_ and *GtXr* could be used to ascertain a gorilla's current group and potential position. The factor (*Fr*) is, in the range [0:1] and characterizes the possibility of deciding on a migrating method to an unsettled location. The *LB* and *UB* are the variables ' minimum and maximum bounds. the variables *D* and *Q* could be determined mathematically by Eqs. (11)–(14). The maximum and present iteration number could be characterized by (*Itn*) and (*MxItn*), respectively. Besides, the symbol (*Z*) is $[-(D\times \left(1-Itn/MxItn\right)),D\times \left(1-Itn/MxItn\right)]$, while the symbol (*s*) is random values among [−1:1].

#### Exploitation stage

Two tactics in this stage are proposed when the factor $D\times \left(1-Itn/MxItn\right)$ is compared with the variable (*Y*). These two behaviors are the escorting the silverback and the competing for adult females. The first one is determined when the value of *Y* equals/ (is less than) the value of $D\times \left(1-Itn/MxItn\right)$, the tactic of the silverback could be selected that can directs the others to food sources. This tactic can be represented mathematically as signified in (15) as follows:

15 $$GtX(Itn+1)=Q\times R(Itn)\times (X(Itn)-{X}_{sb})+X(Itn)$$

16 $$R(Itn)={\left({\left|(1/NG){\sum }_{i=1}^{NG}Gt{X}_{i}(Itn)\right|}^{{2}^{Q}}\right)}^{(\frac{1}{{2}^{Q}})}$$

where *NG* is the gorillas’ population; *X*_*sb*_ indicates the silverback (best solution); *X(Itn)* is the gorilla location vector; *GtX*_*i*_ *(Itn)* signifies the gorilla position o in each iteration *Itn*.

If the value of *Y* is more than the term $D\times \left(1-Itn/MxItn\right)$, the tactic of competing for adult females is selected^38^. This tactic can be represented mathematically as signified in (17) as follows:

17 $$GX(Itn)={X}_{sb}-({X}_{sb}\times L-X(Itn)\times L)\times A,$$

18 $$L=2\times r{n}_{5}-1$$

19 $$A=\beta \times E, E=\left\{\begin{array}{c}N{G}_{1}rn\ge 0.5\\ N{G}_{2}rn<0.5\end{array}\right.$$

where *L* is the force of impact; *rn*_*5*_ is random number from [0:1]; *β* is pre-optimization value which is specified and set to 3; The factor (*A*) vector is the violence level in a fight; and *E* is employed as imitator for the violence efficacy.

The *GtX(Itn)* solution will replace *X(Itn)* if the fitness value of *GtX(Itr)* is less than *X(Itn)*.

#### Improved GTT incorporating tangent flight strategy

**Figure 2:**
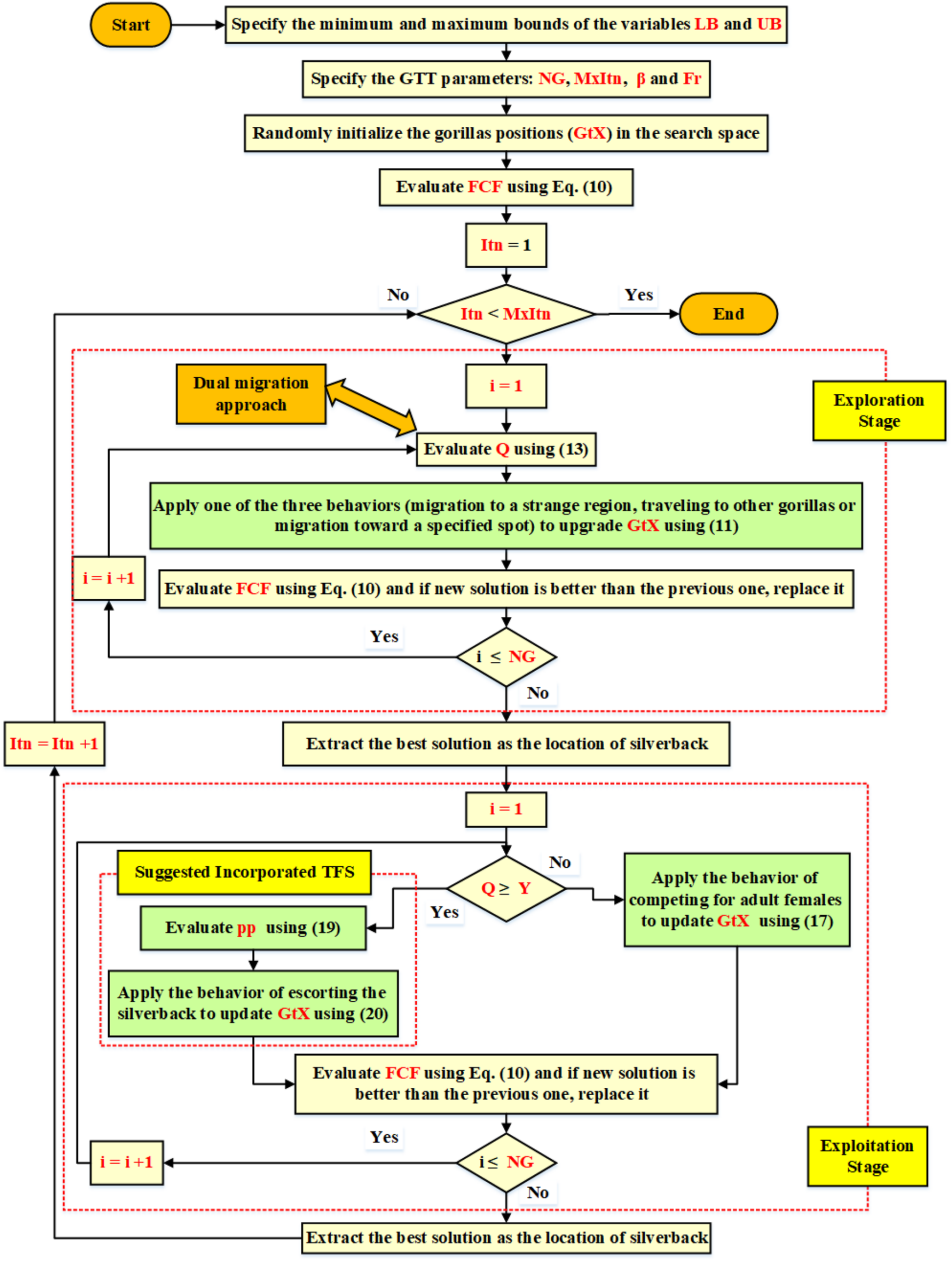
Steps of the IGTT for identifying the FC unknown parameters.

In this part, an improved version of the GTT (IGTT) incorporating Tangent Flight Strategy (TFS). The Cauchy is computed as follows, and its tangent function is the same for the TFS^44^:

20 $$f=\mathit{tan}\left(pp\times \frac{\pi }{2}\right),$$

21 $$pp=randn(1,Dim)$$

where *pp* is a uniformly distributed arbitrary number with a value in the interval [0, 1], and *Dim* is the number of dimensions in the function. This operation is capable of efficiently searching the search space. This function is periodic, and it does not break the balance between both exploration and exploitation. The TFS is added to Eq. (15) by the suggested IGTT approach. The gorilla and silverback's separation will narrow as a result of this modification, drastically reducing the ultimate step size and improving the objective value. This model may be explained mathematically as follows:

22 $$GtX(Itn+1)=(\frac{\mathrm{tan}(\pi \times \frac{2pp-1}{2})}{100})\times Q\times R(Itn)\times (X(Itn)-{X}_{sb})+X(Itn)$$

where *pp* is evaluated using Eq. (19). The key steps for the proposed IGTT are illustrated as depicted in Fig. 2^36^. As shown, the five behaviors in optimizing the gorillas are highlighted in green.

## Simulation results and discussion

Firstly, a comparative assessment of the developed IGTT and the original GTT are demonstrated on the ten common, and well-known benchmark mathematical models following to the Congress on Evolutionary Computation (CEC) 2017 unconstrained benchmark functions^45^. Their mathematical objective model, dimensions, ranges of the control variables and their optimal objective value are announced in Table 1. The first function (F1) represents a unimodal function while the second one (F2) is a multimodal function. The functions (F3–F6) represent mixed functions, and the functions (F7–F10) are composite functions.

**Table 1:** Detailed definition of the ten common, well-known mathematical models under consideration.

Function No.	Optimal	Min	Max	Dim	Function
F1	300	−100	100	30	Shifted and rotated Zakharov function
F2	600	−100	100	30	Shifted and rotated expanded Scaffer’s F6 function
F3	1100	−100	100	30	Hybrid function 1 (N = 3)
F4	1600	−100	100	30	Hybrid Function 6 (N = 4)
F5	1700	−100	100	30	Hybrid function 6 (N = 5)
F6	1900	−100	100	30	Hybrid function 6 (N = 5)
F7	2100	−100	100	30	Composition function 1 (N = 3)
F8	2400	−100	100	30	Composition function 4 (N = 4)
F9	2500	−100	100	30	Composition function 5 (N = 5)
F10	2700	−100	100	30	Composition function 7 (N = 6)

**Figure 3:**
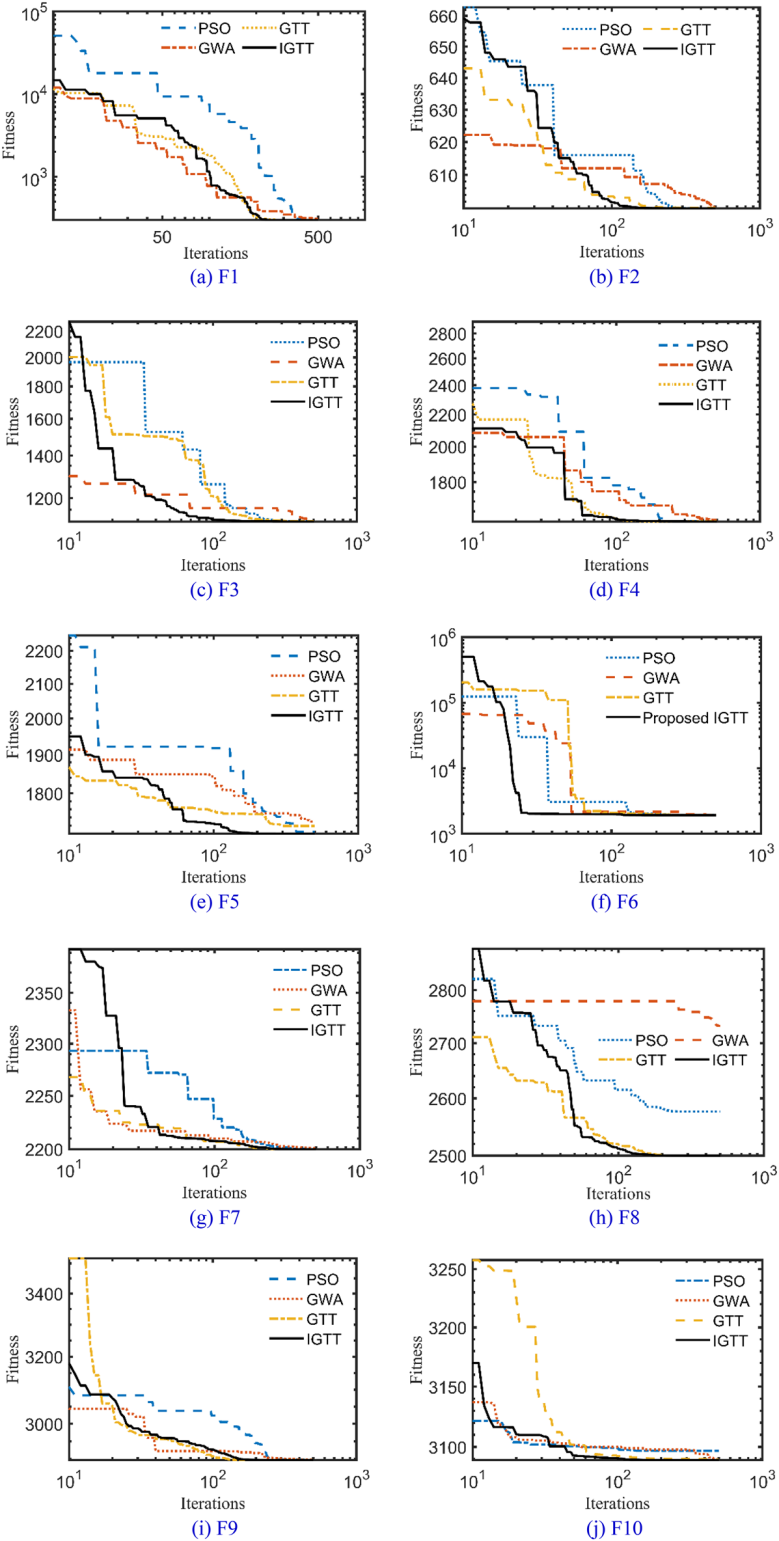
Best convergence curves of the IGTT, GTT, PSO and GWA for benchmarks.

Table 2 shows the performance study of the developed IGTT and the original GTT for ten common, well-known mathematical models and how it compares to two well-established, and well-known optimizers such as GWA^46^ and particle swarm optimization (PSO)^47^. Also, the best regarding convergence characteristics are displayed in Fig. 3. This Table clearly shows that the IGTT performs and operates more effectively than the original GTT, PSO and GWA in the tested mathematical functions, demonstrating the robustness of IGTT in finding the best answer to these mathematical functions. From this Table, the suggested IGTT outperforms the standard GTT in 92.5% of the statistical indices of the investigated benchmark functions for the best, mean, worst, and standard deviations. Similarly, compared to the PSO, the developed IGTT outperforms it in 92.5% of the statistical indices of the investigated benchmark functions. Compared to the GWA, the IGTT outperforms it in 87.5% of the statistical indices of the investigated benchmark functions.

**Table 2:** Performance study of the IGTT, GTT, PSO and GWA for ten common, well-known mathematical models.

Function	Index	Algorithms				IGTT vs PSO	IGTT vs GWA	IGTT vs GTT
		PSO	GWA	GTT	Proposed IGTT			
F1	Best	300.06	310.48	300.00	300.00	1	1	0
	Mean	1578.38	3268.19	300.00	300.00	1	1	1
	Worst	20,019.47	15,183.27	300.00	300.00	1	1	1
	STD	3546.87	2860.15	0.00	0.00	1	1	1
F2	Best	600.00	600.07	600.18	600.00	0	1	1
	Mean	603.45	601.81	608.56	602.07	1	0	1
	Worst	616.02	609.28	632.64	611.74	1	0	1
	STD	4.08	2.02	6.03	2.37	1	0	1
F3	Best	1103.325	1106.808	1103.007	1101.995	1	1	1
	Mean	1205.218	1157.378	1127.185	1111.416	1	1	1
	Worst	1816.178	1377.781	1173.228	1127.875	1	1	1
	STD	124.8757	56.02409	17.68361	6.631911	1	1	1
F4	Best	1601.427	1607.329	1601.428	1600.738	1	1	1
	Mean	1704.993	1750.189	1706.592	1671.014	1	1	1
	Worst	1860.675	2155.505	1975.837	1856.503	1	1	1
	STD	72.15404	129.1761	107.5027	78.0683	0	1	1
F5	Best	1704.609	1729.231	1719.061	1700.8	1	1	1
	Mean	1761.116	1765.681	1743.249	1736.032	1	1	1
	Worst	1866.902	1870.742	1782.644	1758.831	1	1	1
	STD	34.91874	31.83496	13.93582	12.29503	1	1	1
F6	Best	1917.35	1920.302	1908.523	1905.111	1	1	1
	Mean	14,688.71	9463.822	1963.368	1935.025	1	1	1
	Worst	91,792.57	23,815.15	2078.074	2027.433	1	1	1
	STD	17,861.23	7582.319	47.48994	35.91983	1	1	1
F7	Best	2200.001	2200.933	2200	2200	1	1	1
	Mean	2316.145	2304.802	2217.995	2203.876	1	1	1
	Worst	2355.174	2339.213	2320.622	2316.188	1	1	1
	STD	43.17469	37.30524	39.8798	16.27022	1	1	1
F8	Best	2576.574	2732.078	2500	2500	1	1	1
	Mean	2770.463	2749.426	2686.881	2647.925	1	1	1
	Worst	2818.748	2780.715	2789.163	2766.639	1	1	1
	STD	40.19088	13.31095	112.488	123.906	0	0	0
F9	Best	2898.674	2898.193	2897.81	2897.743	1	1	1
	Mean	2962.524	2942.153	2926.967	2927.1	1	1	0
	Worst	3019.521	3030.581	3024.302	2950.618	1	1	1
	STD	37.59257	20.67387	27.90874	23.25528	1	0	1
F10	Best	3096.771	3089.544	3089.738	3089.297	1	1	1
	Mean	3116.86	3100.511	3096.373	3092.899	1	1	1
	Worst	3195.082	3203.113	3107.354	3099.196	1	1	1
	STD	22.70682	17.39673	4.171774	2.837842	1	1	1

1 indicates the advantage of the proposed IGTT while 0 indicates equality or disadvantage.

After that, two instances of common commercial PEMFCs stacks are discussed: the BCS 500-W and the Modular SR-12 PEM units in this study to manifest the performances of the developed IGTT to obtain parameter extraction of FC. In addition to this, different new recently inspired optimizers are implemented for comparative validation which are the original GTT, SDO, FFO and RFO. The compared techniques are performed in the MATLAB environment (MATLAB 2017b) using PC with Intel(R) Core(TM) i7-3632QM CPU @ 2.20 GHz and 8 GB RAM. For fair comparisons, similar circumstances are taken into considerations with 50 solutions as a population size and 100 iterations as a maximum number. It is commonly known that meta-heuristics have a high level of randomness. As a result, the exhibited minimal SSE results are obtained after 100 separate executions, as well as efficiency measurements to verify the correctness of the metrics set by: STD, maximum, and mean values. Table 3 displays the practical boundaries for the unidentified parameters for BCS 500 W and Modular SR-12.

**Table 3:** Datasheets for BCS 500W and modular SR-12 with the practical boundaries for the unidentified parameters.

Stack type	Technical specifications		Practical boundaries		
	BCS 500 W	Modular SR-12	Parameter	Min	Max
*N*	32	48	*ξ*_*1*_ (V)	−1.1997	−0.8532
*L* (µm)	178	25	*ξ*_*2*_.10^–3^ (V/K)	1	5
*A*_*m*_ (cm^2^)	64.0	62.5	*ξ*_*3*_.10^–5^ (V/K)	3.6	9.8
*J*_*m*_ (A/cm^2^)	0.469	0.672	*ξ*_*4*_*.*10^–5^ (V/K)	−26.00	−9.54
*T*_*c*_ (K)	333	323	*Λ*	10	23
*P*_*H2*_ (atm)	1	1.47628	*R*_*c*_ (mΩ)	0.1	0.8
*P*_*O2*_ (atm)	0.2095	0.20950	*Β* (V)	0.0136	0.5000

### Test case 2: BCS 500 module

For the first model, based on the American Company BCS Technologies as the main manufacturer, the BCS 500 W PEMFC stack is considered where the maximum current represents 30 A, and its rated power represents 500 W^48^. The developed IGTT, the original GTT, SDO, FFO and RFO are performed to obtain the best parameter extraction for this model. Table 4 records their best obtained values and regarding SSE objective are illustrated besides, it tabulates the achieved parameters and the accompanying obtained SSE objectives using different published outcomes of recently inspired optimizers. The compared techniques are shuffled multi-simplexes search (SMS) algorithm^2^, WOA^18^, ant lion optimizer (ALO)^27^, GOA^27^, multi-verse optimizer (MVO)^27^, SSO^43^, sine STA (STSA)^49^, MRFO^49^, equilibrium optimization (EO)^49^, Improved Heap-based optimizer (IHBO)^49^, HHO^50^, atom search optimizer (ASO)^50^, moth-fame optimizer (MFO)^51^, SSO^52^, modified HHO (MHHO)^53^, fractional-order MHHO (FMHHO)^53^, vortex search algorithm (VSA)^54^ and VSA and differential evolution (VSDE)^54^.

**Table 4:** Extracted parameters using the proposed IGTT, recent, and reported optimizers for the BCS 500W stack.

Technique	*ξ*_*1*_ (V)	*ξ*_*2*_*.*10^–4^ (V/K)	*ξ*_*3*_*.*10^–5^ (V/K)	*ξ*_*4*_*.*10^–4^ (V/K)	*Λ*	*R*_*c*_ (mΩ)	*β* (V)	SSE 10^–2^
Proposed IGTT	−1.16935	3.1477E−03	3.7232	−1.93017	20.8767777	0.1	1.612586	1.16977808
GTT	−0.85346	2.18019E−03	3.60104	−1.93009	20.8775	0.100487	1.61243	1.1698187
FFO	−0.99989	3.35984E−03	8.36833	−1.93613	21.9056	0.235443	1.55926	1.6297316
SDO	−1.14187	3.57691E−03	7.02680	−1.92532	21.2659	0.139594	1.62312	1.1905119
RFO	−1.02354	3.18392E−03	6.75645	−1.93501	22.3874	0.336282	1.56605	2.1226738
SMS^2^	−0.95250673	51.7616193	5.17616193	−0.9540000	12.57433080	0.10000	1.3600	1.69778
WOA^18^	−1.19693	31.800	3.6000	−1.7700	22.974	0.100	2.216	37.273
ALO^27^	−1.1880	36.840	6.8200	−1.9000	22.5552	0.290	1.60	1.190
GOA^27^	−0.8550	30.320	9.0600	−1.9000	21.0423	0.319	1.46	1.710
MVO^27^	−1.1396	31.910	4.5800	−1.9000	20.5547	0.410	1.41	2.130
SSO^43^	−0.9719	33.487	7.9111	−0.95435	13.0000	0.10000	5.34	1.219
IHBO^49^	−1.19970	33.100	4.2000	−1.9300	20.877	0.100	1.613	1.170
EO^49^	−1.09072	33.300	6.4200	−1.9100	22.177	0.267	1.649	1.462
MRFO^49^	−1.11262	30.600	4.2300	−1.9500	21.705	0.111	1.718	3.683
STSA^49^	−0.85320	21.800	3.8300	−1.9100	18.062	0.100	1.383	2.135
HHO^50^	−1.09311	32.8141	5.67397	−1.89666	20.0436	0.225793	151.48	1.4879
ASO^50^	−1.0432	36.745	8.8772	1.8775	23.3295	0.581379	1.6495	2.661
MFO^51^	−1.0079	33.230	7.9800	−1.9000	20.9189	0.154	1.58	1.190
SSO^52^	−1.0074	33.470	8.1500	−1.9000	18.9165	0.121	1.50	1.610
FMHHO^53^	−0.87884	30.236	8.2272	−1.1934	22.709	0.40472	1.5289	1.1770
MHHO^53^	−0.91048	30.661	7.9053	−1.9098	19.384	0.10320	1.5212	1.3511
HHO^53^	−0.96053	33.505	8.7377	−1.8967	21.821	0.42358	1.5006	1.5753
VSDE^54^	−1.1970	42.330	9.7990	−1.9201	20.194	0.1108	1.57	1.214
VSA^54^	−1.0005	3.0053	5.8273	−1.9498	22.322	0.2161	1.58	1.570

From this table, the developed IGTT derives the best performance with the smallest SSE objective value of 0.0016977 compared to GTT, SDO, FFO and RFO. The original GTT achieves SSE value of 0.0116982 where FFO, SDO and RFO obtain SSE values of 0.0119, 0.0163 and 0.0212, respectively. Moreover, compared to published results, the developed IGTT declares very high outperformance over WOA^18^, GOA^27^, MVO^27^, SSO^43^, STSA^49^, MRFO^49^, EO^49^, HHO^50^, ASO^50^, SSO^52^, MHHO^53^, HHO^53^ and VSA^54^. Also, the GTT shows a significant supremacy compared to ALO^27^, MFO^51^ and VSDE^54^ which obtain SSE objectives of 0.0119, 0.0119 and 0.01214, respectively. Additionally, the developed IGTT shows a small comparable preponderance compared to SMS^2^, IHBO^49^ and FMHHO^53^ which obtain SSE objectives of 0.0169778, 0.0117 and 0.01177, respectively.

**Figure 4:**
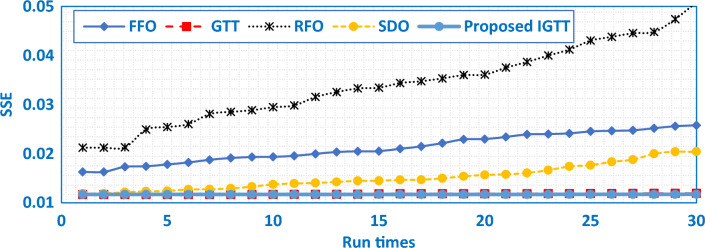
Sample of the best 30 runs of the proposed IGTT, GTT, SDO, FFO and RFO for BCS 500W stack.

To contrast the robustness validation of the developed IGTT with GTT, SDO, FFO and RFO, Fig. 4 describes the best SSE values of a 30 run times sample. As shown, the relative optimum SSE values are related to the developed IGTT where the attained results by the developed IGTT always supersede the GTT, SDO, FFO and RFO as represented in that figure. Not only that, but Table 5 illustrates their comparative assessment for the BCS 500W Stack with several other published results through the best, mean, worst and STD over the separate runs. As shown, the proposed IGTT has the best effectiveness since it acquires the least good, mean, worst and STD values of 0.011697781, 0.014329, 0.02699 and 0.0053594, respectively.

**Table 5:** Comparative assessment of the proposed IGTT with reported optimizers for the BCS 500W stack.

Technique	*Best*.(10^–2^)	*Mean*.(10^–2^)	*Worst*.(10^–2^)	*STD*
Proposed IGTT	1.16977808	1.4329	2.699	0.005359
GTT	1.1698187	2.6388	7.0539	0.016382
FFO	1.6297316	3.4934	10.8863	0.017344
SDO	1.1905119	5.9894	36.3407	0.071129
RFO	2.1226738	23.9040	153.8797	0.308101
WOA^18^	37.273	256.3700	851.7320	2.55770
ALO^27^	1.1900	20.6000	60.5200	0.1880
GOA^27^	1.7100	43.8549	221.0571	67.4693
MVO^27^	2.1300	5.2500	13.5600	0.1565
STSA^49^	2.1350	62.114	340.1770	0.70331
MRFO^49^	3.6830	39.1070	113.428	0.25386
EO^49^	1.4620	4.6460	13.4140	0.03633
MFO^51^	1.1900	4.7800	13.5100	0.0434
SSA^52^	1.6100	16.2300	47.8000	0.1565
FMHHO^53^	1.7700	37.3710	13.4980	0.13008
MHHO^53^	1.3511	24.0670	55.45000	0.15958
HHO^53^	1.5753	28.2000	42.2330	0.13165

**Figure 5:**
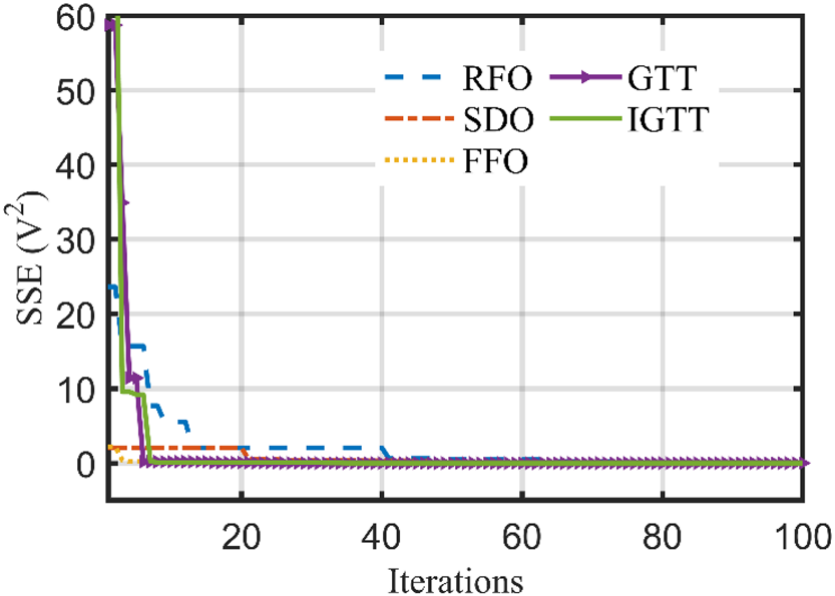
Best convergence patterns of the IGTT, GTT, SDO, FFO and RFO for BCS 500W stack.

Also, Fig. 5 depicts the best convergence curves related to the IGTT, GTT, SDO, FFO and RFO for BCS 500W Stack. As shown, the IGTT has the fastest response in finding the minimum SSE in approximately 30% of the iteration’s axis.

**Figure 6:**
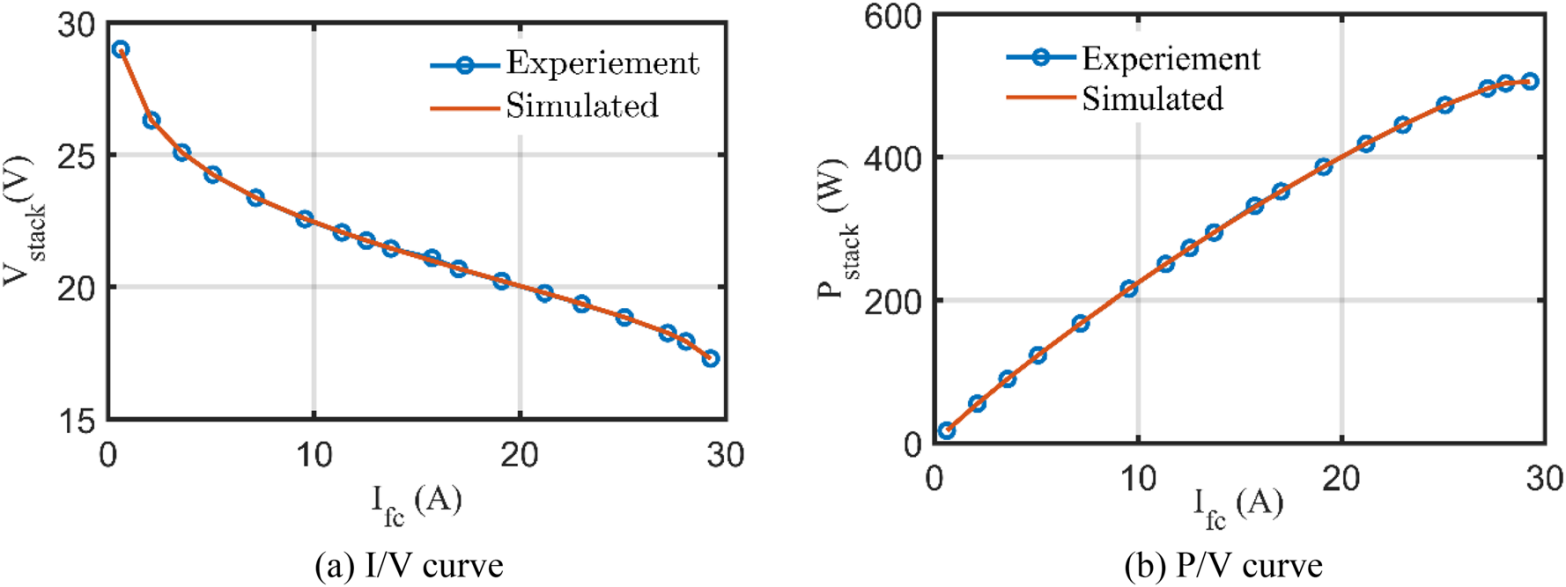
I/V and P/V curves based on the IGTT’s parameters extraction for BCS 500W stack.

Based on the IGTT parameters extraction for BCS 500W Stack, Fig. 6 shows the regarding I/V and P/V characteristics compared to the related experimental recordings (The regarding values are tabulated in the appendix, kindly refer to Table A.1). As shown, excellent fittings among the simulated and measured I/V and P/V characteristics are observed.

**Figure 7:**
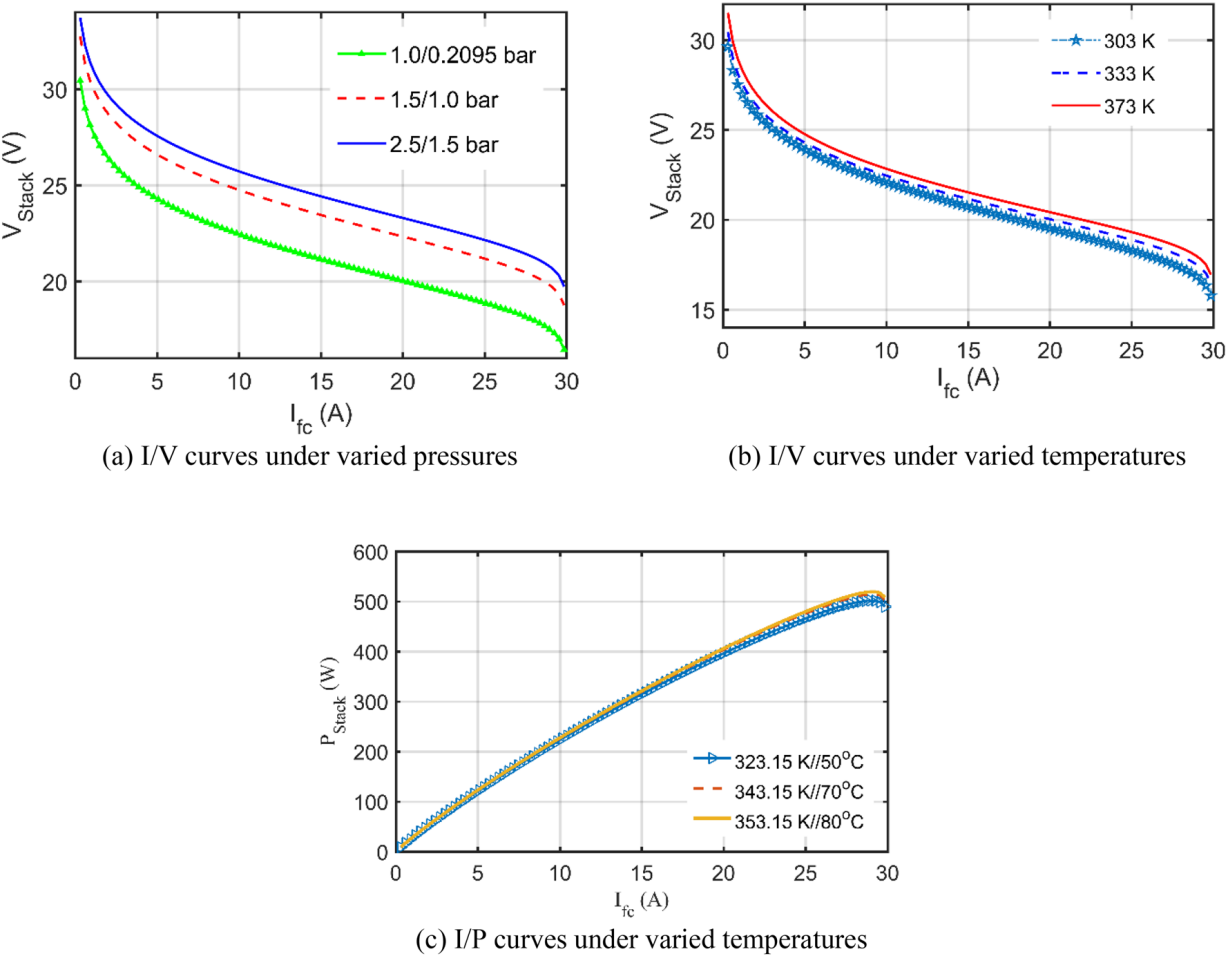
Performance curves based on the IGTT’s parameters extraction for BCS 500W stack under varied conditions.

In contrast, the described polarization characteristics in terms of I/V and I/P plots are shown in Fig. 7a–c. First, the I/V curves are plotted under the pressures of ${\mathrm{P}}_{{\mathrm{H}}_{2}}/{\mathrm{P}}_{{\mathrm{O}}_{2}}$ of 1.000/0.2095 bar, 1.5/1.0 bar, and 2.5/1.5 bar; respectively, at a constant cell temperature of 333 K which are shown in Fig. 7a. Then, the temperature’s variations are simulated at 303 K, 333 K and 373 K; respectively at constant partial pressures as specified in the datasheet (i.e. ${\mathrm{P}}_{{\mathrm{H}}_{2}}/{\mathrm{P}}_{{\mathrm{O}}_{2}}$=1.0/0.2095) which are depicted in Fig. 7b. In addition to that, the I/P curves are plotted under varied temperatures at 60 °C, 70 °C, and 80 °C, respectively as depicted in Fig. 7c. These curves are exceptionally smooth under various operating situations, offering confidence in the IGTT-based model's high efficiency.

### Test case 2: modular SR-12

The parameter extraction algorithms are thoroughly validated using the Modular SR-12 PEMFCs to verify how well the IGTT based-parameter extraction approach performs. The IGTT, GTT, SDO, FFO and RFO are performed to obtain the best parameter extraction for this model where their best obtained values and regarding SSE objective are tabulated in Table 6. In addition to this, different published outcomes of recently inspired optimizers are added in this table such as WOA^18^, flower pollination algorithm (FPA)^30^, MRFO^49^, EO^49^, STSA^49^, MFO^51^, SSO^52^ and interior search algorithm (ISA)^55^.

**Table 6:** Extracted parameters using the GTT, recent, and reported optimizers for Modular SR-12.

Technique	*ξ*_*1*_ (V)	*ξ*_*2*_*.*10^–4^ (V/K)	*ξ*_*3*_*.*10^–5^ (V/K)	*ξ*_*4*_*.*10^–4^ (V/K)	*λ*	*R*_*c*_ (Ω)	*Β* (V)	SSE.10^–4^
Proposed IGTT	−0.93739	27.52	5.208	−1.06348	21.56945	0.0002728	0.1500116	1.421011
GTT	−0.86278	24.3984	4.68307	−1.06348	21.58254	0.000273	0.150013	1.421011
FFO	−0.89444	23.7241	3.60000	−1.06862	19.22577	0.00032	0.148312	6.900092
RFO	−0.86334	27.1255	6.44383	−1.06781	13.72355	0.00011	0.148234	8.619470
SDO	−0.97879	30.8768	6.55143	−1.06150	21.90073	0.000298	0.149725	3.637125
WOA^18^	−1.19970	42.7	9.78	−1.08	18.83208	0.119	0.15125	20.2
FPA^30^	−0.85320	31.0	9.15	−9.54	13.0000	0.571	0.14548	1598.2
MRFO^49^	−1.15570	36.5	6.62	−1.05	20.54620	0.224	0.15288	625.6
EO^49^	−1.08514	31.8	5.03	−1.06	20.39017	0.337	0.14874	12.0
ISA^55^	−1.16993	36.6	6.45	−1.07	13.92287	0.112	0.14915	1.9
SSO^52^	−1.03331	37.2	9.57	−9.58	14.30103	0.799	0.14212	968.1
MFO^51^	−1.12876	39.5	9.13	−1.00	20.14571	0.800	0.14274	433.4
STSA^49^	−0.85320	22.4	3.60	−1.06	13.0000	0.100	0.14856	8.7

**Figure 8:**
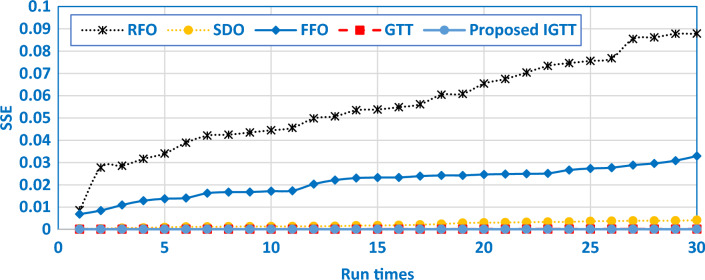
Sample of the best 30 runs of the GTT SDO, FFO and RFO for modular SR-12.

As demonstrated, when compared to SDO, FFO, and RFO, the IGTT besides GTT have the capability to achieve the best performance with the smallest SSE target. Additionally, the formed IGTT claims the best performance with the least SSE when compared to published findings. The IGTT with GTT, SDO, FFO, and RFO's best SSE values from a sample of 30 runs are shown in Fig. 8 for the robustness comparison. The relative optimum SSE values are related to the IGTT, where the IGTT's obtained results always take precedence over the GTT, SDO, FFO, and RFO given in that figure. Additionally, Table 7 compares their evaluation of the Modular SR-12 Stack with several other published results using the best, mean, worst, and STD across many runs. As evident, the IGTT has the best effectiveness since it acquires the least good, mean, worst and STD values of 1.421E-4, 19.593E-4, 281.22E-4 and 5.277E-3, respectively.

**Table 7:** Comparison of the IGTT with reported optimizers for Modular SR-12 stack.

Technique	*Best.*(10^–4^)	*Mean.*(10^–4^)	*Worst.*(10^–4^)	*STD*
Proposed IGTT	1.421011	19.593	281.22	5.2776 E−3
GTT	1.421	37.144	395.870	7.1727E−3
SDO	3.637	200.940	5239.60	5.4800E−2
FFO	6.900	879.720	6600.400	9.5520E−2
RFO	8.619	4209.200	68,075.000	9.5157E−1
FPA^30^	1589.200	11,575.500	51,239.500	1.04838
STSA^49^	8.700	1000.200	3351.300	0.09099
EO[ 43]	12.000	195.200	739.600	0.02002
MRFO^49^	625.600	7872.700	40,135.500	0.89801
MFO^51^	433.400	1321.600	3518.400	0.05970
SSA^52^	968.100	1802.800	3663.000	0.08660

**Figure 9:**
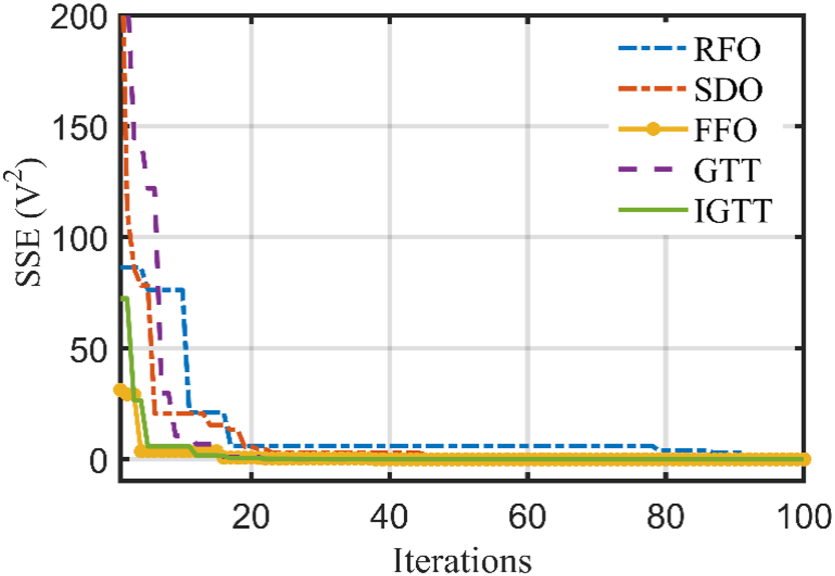
Best convergence curves of the IGTT, GTT, SDO, FFO and RFO for modular SR-12.

Additionally, Fig. 9 shows the IGTT, GTT, SDO, FFO, and RFO for Modular SR-12 stack's finest convergence properties. As demonstrated, the zooming part in this figure is dedicated for illustrating the capability of the IGTT and GTT in faster reaching the optimal solution after only 35% of the iteration journey while the SDO approaches to a very close value after 92% of the journey. On the other side, RFO and FFO fails to achieve a close value through 100% of the iteration journey.

**Figure 10:**
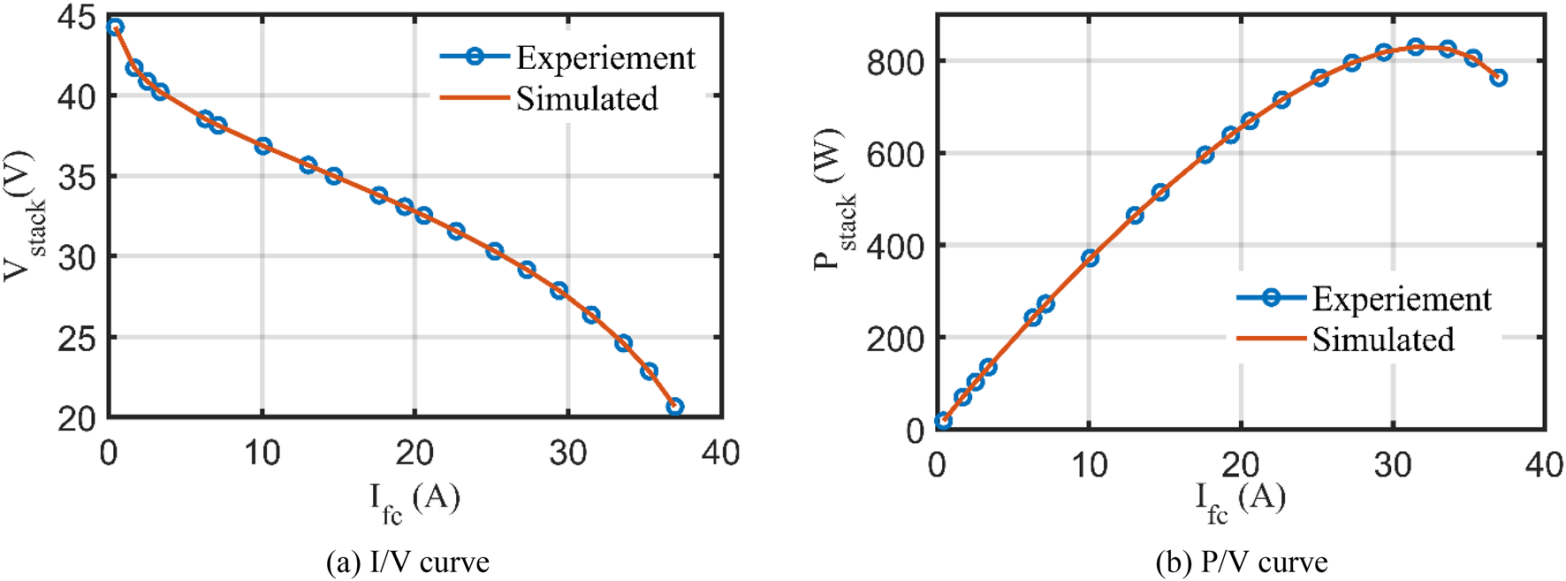
I/V and P/V curves based on the IGTT parameters extraction for modular SR-12.

**Figure 11:**
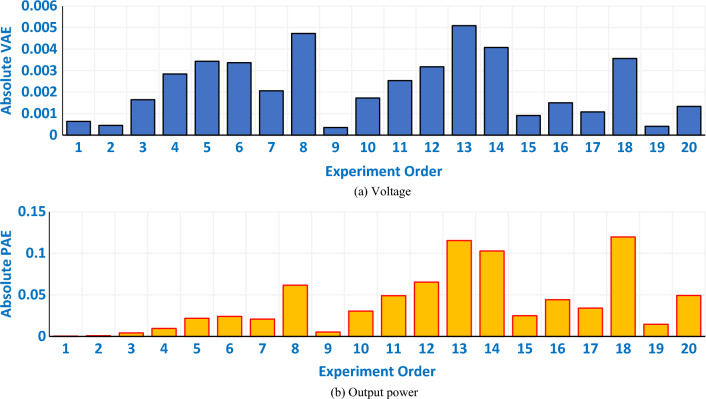
Absolute errors of the voltage and output power based on the IGTT parameters extraction for modular SR-12 stack.

Interestingly, Fig. 10 depicts the relevant I/V and P/V curves in comparison to the relevant experimental recordings based on the IGTT’s parameters extraction for Modular SR-12 Stack. The generated and measured I/V and P/V curves provide excellent fits, as seen. Confirmation of this, Fig. 11 displays the regarding absolute errors between the experimental and the simulated curves (The regarding values are tabulated in the appendix, see Table A.2). It can be noticed that the maximum error of the voltage data points is lesser than 0.016% while the maximum error of the power data points is lesser than 0.015%.

## Conclusion

In this article, an advanced IGTT incorporating a Tangent Flight Strategy (TFS) within the exploitation stage has been employed to effectively extract the PEMFC model's unidentified parameters. A precise model of the PEMFCs is created via the IGTT that delivers accurate simulation and modelling results for two industrial FCs with type of BCS 500W and Modular SR-12 Stacks. Through the IGTT development, the output voltage model of FC's total squared error between the measured and its optimally estimated is minimized for both PEMFCs stacks. Following the CEC 2017, a comparison of the established IGTT and the original GTT is performed against ten unrestricted benchmark functions. In 92.5%, 87.5%, and 92.5% of the statistical indices, the suggested IGTT beats the traditional GTT, the GWA and the PSO. Computer simulations are used to show the robustness of the PEMFC’s model across a range of temperature and pressure variations. By contrasting the numerical modeling outcomes against the experimental findings of the commercial PEMFCs stacks under study, the suggested model's effectiveness is assured. For each case study, the simulated results based on the IGTT are compared to several new optimization techniques of SDO, FFO and RFO. The employed IGTT provides higher accuracy and robustness compared with recently techniques of SDO, FFO, and RFO. Additionally, the outcomes from IGTT are contrasted with those from other optimization techniques. The outcomes and statistical assessments manifest the IGTT superiority compared with several previously reported results which demonstrate its promising features in defining the PEMFC’s model parameters. This leads to the IGTT-based model having a significant advantage over other optimizing method-based models from the literature. Consequently, the IGTT application can offer a precise PEMFC’s model.

Based on the successful application of the IGTT for PEMFC modules parameter estimation in this paper, as a future research trend, the IGTT algorithm is recommended to be employed to solve further advanced engineering problems especially in power systems such as controllers design for power system stability including renewable sources, battery models identification, optimal operation of power systems with renewable sources penetrations.

## Supplementary Information

Supplementary Information.

## Data Availability

The data that support the findings of this study are available from the corresponding author upon reasonable request.
