# Supplementary material for: Fuel-cell parameter estimation based on improved gorilla troops technique
Source: Sci Rep. 2023 May 29;13:8685. doi: 10.1038/s41598-023-35581-y (PMC10227001; doi:10.1038/s41598-023-35581-y)
Supplement: Supplementary file 1 — Supplementary Information. [file 41598_2023_35581_MOESM1_ESM.docx]

**Appendix**

Tables A.1 and A.2 provide the experimental I-V, P-V and the regarding absolute errors in the simulated voltage and power for the BCS 500 W and Modular SR-12, respectively.

Tables A.1: Experimental I-V, P-V and the absolute errors in the simulated voltage and power for the BCS 500 W by IGTT.

| Experimental Order | Experimental Current | Experimental Voltage | Simulated Voltage | Experimental Power | Simulated Power | Absolute Error in Voltage | Absolute Error in Power |
| --- | --- | --- | --- | --- | --- | --- | --- |
| 1 | 0.60 | 29.00 | 28.997 | 17.4000 | 17.39833 | 0.0027827 | 0.0016696 |
| 2 | 2.10 | 26.31 | 26.306 | 55.2510 | 55.24246 | 0.004066 | 0.0085385 |
| 3 | 3.58 | 25.09 | 25.094 | 89.8222 | 89.83492 | 0.0035527 | 0.012719 |
| 4 | 5.08 | 24.25 | 24.255 | 123.1900 | 123.2135 | 0.0046175 | 0.023457 |
| 5 | 7.17 | 23.37 | 23.375 | 167.5629 | 167.6017 | 0.0054124 | 0.038807 |
| 6 | 9.55 | 22.57 | 22.585 | 215.5435 | 215.683 | 0.01461 | 0.13953 |
| 7 | 11.35 | 22.06 | 22.071 | 250.3810 | 250.5095 | 0.011321 | 0.1285 |
| 8 | 12.54 | 21.75 | 21.758 | 272.7450 | 272.851 | 0.0084566 | 0.10605 |
| 9 | 13.73 | 21.45 | 21.461 | 294.5085 | 294.663 | 0.011255 | 0.15453 |
| 10 | 15.73 | 21.09 | 20.988 | 331.7457 | 330.137 | 0.10227 | 1.6087 |
| 11 | 17.02 | 20.68 | 20.694 | 351.9736 | 352.2204 | 0.0145 | 0.24678 |
| 12 | 19.11 | 20.22 | 20.231 | 386.4042 | 386.6139 | 0.010975 | 0.20973 |
| 13 | 21.20 | 19.76 | 19.771 | 418.912 | 419.1437 | 0.010931 | 0.23174 |
| 14 | 23.00 | 19.36 | 19.366 | 445.2800 | 445.4183 | 0.0060118 | 0.13827 |
| 15 | 25.08 | 18.86 | 18.866 | 473.0088 | 473.1706 | 0.0064533 | 0.16185 |
| 16 | 27.17 | 18.27 | 18.275 | 496.3959 | 496.5238 | 0.004709 | 0.12794 |
| 17 | 28.06 | 17.95 | 17.953 | 503.6770 | 503.7696 | 0.0033009 | 0.092625 |
| 18 | 29.26 | 17.30 | 17.293 | 506.1980 | 505.9895 | 0.0071269 | 0.20853 |

Tables A.2: Experimental I-V, P-V and the absolute errors in the simulated voltage and power for the Modular SR-12 by IGTT.

| Experimental Order | Experimental Current | Experimental Voltage | Simulated Voltage | Experimental Power | Simulated Power | Absolute Error in Voltage | Absolute Error in Power |
| --- | --- | --- | --- | --- | --- | --- | --- |
| 1 | 0.42 | 44.25 | 44.251 | 18.5850 | 18.585 | 0.00063774 | 0.00026785 |
| 2 | 1.68 | 41.71 | 41.71 | 70.0728 | 70.074 | 0.00045028 | 0.00075647 |
| 3 | 2.52 | 40.87 | 40.868 | 102.9924 | 102.99 | 0.0016429 | 0.0041401 |
| 4 | 3.36 | 40.22 | 40.217 | 135.1392 | 135.13 | 0.0028418 | 0.0095483 |
| 5 | 6.30 | 38.53 | 38.533 | 242.739 | 242.76 | 0.003433 | 0.021628 |
| 6 | 7.14 | 38.13 | 38.133 | 272.2482 | 272.27 | 0.0033672 | 0.024042 |
| 7 | 10.08 | 36.85 | 36.852 | 371.4480 | 371.47 | 0.0020624 | 0.020789 |
| 8 | 13.02 | 35.66 | 35.655 | 464.2932 | 464.23 | 0.0047291 | 0.061573 |
| 9 | 14.70 | 34.98 | 34.98 | 514.2060 | 514.2 | 0.00035524 | 0.0052221 |
| 10 | 17.64 | 33.78 | 33.778 | 595.8792 | 595.85 | 0.0017259 | 0.030445 |
| 11 | 19.32 | 33.07 | 33.067 | 638.9124 | 638.86 | 0.0025367 | 0.049008 |
| 12 | 20.58 | 32.52 | 32.517 | 669.2616 | 669.2 | 0.0031768 | 0.065378 |
| 13 | 22.68 | 31.55 | 31.555 | 715.554 | 715.67 | 0.0050864 | 0.11536 |
| 14 | 25.20 | 30.30 | 30.304 | 763.5600 | 763.66 | 0.0040782 | 0.10277 |
| 15 | 27.30 | 29.15 | 29.151 | 795.7950 | 795.82 | 0.00091169 | 0.024889 |
| 16 | 29.40 | 27.86 | 27.858 | 819.0840 | 819.04 | 0.0015032 | 0.044194 |
| 17 | 31.50 | 26.37 | 26.371 | 830.6550 | 830.69 | 0.0010772 | 0.033933 |
| 18 | 33.60 | 24.60 | 24.596 | 826.5600 | 826.44 | 0.0035622 | 0.11969 |
| 19 | 35.28 | 22.86 | 22.86 | 806.5008 | 806.49 | 0.00041185 | 0.01453 |
| 20 | 36.96 | 20.66 | 20.661 | 763.5936 | 763.64 | 0.0013319 | 0.049227 |
